# Comparison of Serum Brain-Derived Neurotrophic Factor Levels in Diabetic Patients With and Without Retinopathy

**DOI:** 10.7759/cureus.12028

**Published:** 2020-12-11

**Authors:** Romana R Rashid, Ayesha Fazal, Qudsia U Khan, Misbah Ul Qamar, Farooq Ahmed, Talha Laique

**Affiliations:** 1 Physiology, Akhtar Saeed Medical and Dental College, Lahore, PAK; 2 Physiology, Combined Military Hospital (CMH) Lahore Medical and Dental College, Lahore, PAK; 3 Ophthalmology, Mayo Hospital, Lahore, PAK; 4 Pharmacology, Lahore Medical and Dental College, Lahore, PAK

**Keywords:** retinopathy, brain derived neurotrophic factor, diabetes type 2

## Abstract

Introduction

Diabetes mellitus (DM) is a disease of carbohydrate, protein, and fats metabolism that results in hyperglycemia. It may also result from defects in the secretion of insulin from beta cells or in its action on target cells or both.

Objective

To determine the levels of brain-derived neurotrophic factor (BDNF) and glycated hemoglobulin (HbA1c) with the progression of retinopathy.

Methodology

The study was done on 80 patients who were divided into four groups (A, B, C, D), with 20 individuals each, on the basis of their diabetic status and fundoscopic findings. Serum BDNF levels were measured by using an enzyme-linked immunosorbent assay kit (Glory Science Co., Taichung City, Taiwan).

Results

On analysis, a significant decline was seen in serum BDNF levels in diabetics as compared with non-diabetics (p < 0.001), but a significant reduction in levels with the progression of retinopathy was observed (p < 0.001).

Statistical analysis

All the data were processed using the Statistical Package for the Social Sciences (SPSS) v20.0 (IBM Corp., Armonk, NY).

Conclusion

There is a significant decline in serum BDNF levels in type 2 diabetics with retinopathy in comparison with the healthy control group.

## Introduction

Diabetes mellitus (DM) is a disease that affects the metabolism of carbohydrates, proteins, and fats, which results in hyperglycemia. It may result from defects in the secretion of insulin from beta cells or in its actions on target cells or both [1]. According to the diabetic prevalence survey (DPSP), which was conducted in 2017 in both rural and urban areas, the number of pre-diabetics is rising all over Pakistan. It suggested that the prevalence of diabetics is in the ratio 1:7 among people older than 20 years of age. The World Health Organization (WHO) ranked Pakistan seventh on the prevalence list [2]. DM may lead to long-term damage to various body organs like kidneys, nerves, and eyes, resulting in their dysfunction and failure.

Diabetes mellitus can present with distinctive symptoms such as polydipsia, polyphagia, polyuria, blurring of vision, weight loss, and hyperglycemia. Uncontrolled diabetes can lead to ketoacidosis or a hyperosmolar state, ultimately resulting in coma or death if not treated effectively [1]. The disease may remain undiagnosed for a long time due to the mildness or absence of symptoms in earlier stages. The resulting hyperglycemia leads to functional and pathological alterations of major body organs before the diagnosis. One of the long-term complications of diabetes is progressive retinopathy with resulting blindness among middle-aged people worldwide. Other long-term complications include nephropathy and neuropathy. Nephropathy leads to renal failure while neuropathy increases the threat of foot ulcers, which may lead to amputation, autonomic disorders like sexual dysfunction, and Charcot joints [3].

Insulin helps in the control of blood glucose levels by promoting the uptake of glucose by target cells, which includes skeletal muscles, the liver, and fatty tissues after taking meals [4]. Persistent hyperglycemia intrudes on the regulation of many neurotrophic and growth factors in the eye and leads to neuroinflammation, apoptosis, and glutamate excitotoxicity, aiding progression to retinopathy [5].

Brain-derived neurotrophic factor (BDNF) is a protective factor for neuronal growth, survival, and synaptic plasticity and regulates the synthesis and uptake of neurotransmitters. It is synthesized by neuronal and glial cells as well as by some non-neuronal tissues like immune cells, the vascular endothelium, and platelets. This neurotrophic factor is formed as a pro-neurotrophin, which is then converted into mature BDNF by some proteases. Mature BDNF binds with tyrosine kinase B receptor to activate cellular signals, which promote neuronal differentiation and survival by inhibiting neurodegeneration and angiogenesis by reducing oxidative stress in the retina. Previous studies have highlighted BDNF as a significant factor in the progress of retinopathy [6].

Various studies have shown that BDNF levels are significantly reduced in the serum and retina of diabetic patients [7-8]. However, there is a lack of local data regarding the variation of BDNF levels among diabetic patients with progressive grades of diabetic retinopathy; hence, it remained undiscovered. In light of this increasing burden among our population, we planned the current study to determine the levels of BDNF and glycated hemoglobin (HbA1c) with the progression of retinopathy in the present study.

## Materials and methods

The present study was conducted in the physiology department in collaboration with the diabetic clinic and ophthalmology department of Sheikh Zayed Medical Complex, Lahore, from March 2017 to April 2018. It is a descriptive cross-sectional study. All known type 2 diabetics, having the disease for a duration of a minimum of five years, with age ≥30 years were enrolled throughout the project following approval from the hospital’s ethical committee. Written informed consent was taken from all the patients. Patients with hypertensive retinopathy on fundoscopy, history of epilepsy, autoimmune disease or malignant disease, and cataract or glaucoma on examination were excluded [9]. The sample size consisting of 80 subjects was estimated by keeping a 95% level of confidence as used by one previous study. It was also calculated by using the WHO sample size calculator. Patients were divided into four groups after fundoscopy, with each group consisting of 20 participants.

Group 1 (control group): It consisted of 20 non-diabetics without evidence of retinopathy on fundoscopic examination.

Group 2 (study group): It consisted of 20 diabetics without evidence of retinopathy on fundoscopic examination.

Group 3 (study group): It consisted of 20 diabetics with evidence of non-proliferative retinopathy that is characterized by the presence of micro-aneurysms, hemorrhages, venous beading, hard exudate, and intraretinal microvascular abnormalities on fundoscopic examination.

Group 4 (study group): It consisted of 20 diabetics with evidence of proliferative retinopathy that is characterized by the presence of neovascularization on fundoscopic examination.

Three ml blood was collected for serum BDNF and immediately centrifuged at 3000 rpm for 20 min. The serum was separated and then stored in a refrigerator at a temperature of -20 ° C. The test was accomplished by the enzyme-linked immunosorbent assay (ELISA) technique at the RIA laboratory, National Health Research Complex (NHRC), Lahore.

The normality of the data was tested using the Shapiro-Wilk test. Data were found to be normally distributed. Means among groups were compared using one-way analysis of variance (ANOVA). The chi-square/Fisher’s exact test was applied to determine the link of categorical data with the groups. A p-value of ≤0.05 was considered statistically significant.

## Results

The distribution of different parameters among enrolled groups as mean ± SD are summarized in Table 1.

**Table 1:** Distribution of age, HbA1c, and BDNF among enrolled groups as mean ± SD HbA1c: glycated hemoglobin; BDNF: brain-derived neurotrophic factor

Distribution of age, HbA1c, and BDNF				
Age	Mean ± SD	Minimum (years)	Maximum (Years)	P-Value
Non-diabetics	49.3 ± 5.7	39	59	0.181
Diabetics without Diabetic Retinopathy	51.8 ± 6.4	42	64	
Diabetics with Non-Proliferative Diabetic Retinopathy	53.1 ± 6.0	42	64	
Diabetics with Proliferative Diabetic Retinopathy	53.1 ± 6.9	40	65	
HbA1c	Mean ± SD	Minimum (%)	Maximum (%)	P-Value
Non-diabetics	5.0 ± 0.3	4.5	5.7	< 0.001*
Diabetics without Diabetic Retinopathy	8.7 ± 1.4	6.8	10.9	
Diabetics with Non-Proliferative Diabetic Retinopathy	9.8 ± 1.1	8.1	12.8	
Diabetics with Proliferative Diabetic Retinopathy	10.8 ± 1.2	8.7	13.0	
BDNF	Mean ± SD	Minimum (ng⁄ml)	Maximum (ng⁄ml)	P-Value
Non-diabetics	20.4 ± 0.6	19.3	21.2	< 0.001*
Diabetics without Diabetic Retinopathy	19.2 ± 0.6	18.4	20.1	
Diabetics with Non-Proliferative Diabetic Retinopathy	18.6 ± 0.6	17.5	19.5	
Diabetics with Proliferative Diabetic Retinopathy	17.2 ± 0.6	15.9	18.3	

The percentages and frequencies of qualitative variables like gender, hypertension, and cardiovascular diseases are summarized in Table 2.

**Table 2:** Distribution of qualitative variables as frequency and percentage DR: diabetic retinopathy; NPDR: non-proliferative diabetic retinopathy; PDR: proliferative diabetic retinopathy

Distribution of qualitative variables			
Gender	Male n (%)	Female n (%)	P-Value
Non-diabetics	12 (60.0%)	8 (40.0%)	0.937
Diabetics without DR	13 (65.0%)	7 (35.0%)	
Diabetics with NPDR	11 (55.0%)	9 (45.0%)	
Diabetics with PDR	12 (60.0%)	8 (40.0%)	
Hypertension	Yes	No	P-Value
Non-diabetics	0 (0.0%)	20 (100%)	< 0.001*
Diabetics without DR	11 (55.0%)	9 (45.0%)	
Diabetics with NPDR	15 (75.0%)	5 (25.0%)	
Diabetics with PDR	16 (80.0%)	4 (20.0%)	
Cardiovascular disease	Yes	No	P-Value
Non-diabetics	0 (0.0%)	20 (100%)	0.228
Diabetics without DR	2 (10.0%)	18 (90.0%)	
Diabetics with NPDR	4 (20.0%)	16 (80.0%)	
Diabetics with PDR	3 (15.0%)	17 (85.0%)	

The mean BDNF was significantly greater in the non-diabetics group as compared to the remaining groups as shown in Table 3.

**Table 3:** Pair-wise comparison of BDNF among groups by using Tukey's test BDNF: brain-derived neurotrophic factor; DR: diabetic retinopathy; NPDR: non-proliferative diabetic retinopathy

Pair-wise comparison of BDNF				
Groups	Groups	Mean Difference	Std. Error	P-value
Non-diabetics	Diabetics without Diabetic Retinopathy	1.16345*	.18293	0.000*
	Diabetics with Non-Proliferative Diabetic Retinopathy	1.81475*	.18293	0.000*
	Diabetics with Proliferative Diabetic Retinopathy	3.16250*	.18293	0.000*
Diabetics without DR	Diabetics with Non-Proliferative Diabetic Retinopathy	.65130*	.18293	0.004*
	Diabetics with Proliferative Diabetic Retinopathy	1.99905*	.18293	0.000*
Diabetics with NPDR	Diabetics with Proliferative Diabetic Retinopathy	1.34775*	.18293	0.000*

## Discussion

Diabetes mellitus is a metabolic derangement either due to lack of insulin or resistance to insulin and its resulting hyperglycemia. Visual impairment and blindness due to retinal damage is one of the major complications of diabetes. In the present study, a comparison between the serum levels of BDNF and HbA1c among diabetics with or without retinopathy was done taking non-diabetics as the healthy group. The association of various factors like cardiovascular diseases and hypertension with diabetic retinopathy was also studied [10].

Our study revealed that the history of hypertension in participants was significantly associated with diabetic retinopathy (p-value < 0.001). A study carried out on Indian immigrants in Singapore showed that raised systolic blood pressure was positively associated with the development and progression of diabetic retinopathy [11]. This may be attributed to the reduced retinal blood flow caused by the stiffening of arteries due to hypertension.

Previously, studies have consistently indicated a strong association of cardiovascular diseases with retinal microangiopathies. Ciccacci et al. have shown that the polymorphism of the same gene contributes to diabetic retinopathy and cardiac autonomic neuropathy [12]. However, our study did not indicate the possible role of cardiovascular diseases in the development and progression of diabetic retinopathy (p-value 0.228). This may be due to the fact that the frequency of subjects with cardiovascular diseases in our study was much lower as compared to other studies, resulting in the inability to detect the effect of these conditions on diabetic retinopathy.

In the current study, we measured and compared the serum levels of BDNF among diabetics and non-diabetics, and our data of BDNF measurements in diabetic patients corresponded with previous findings that revealed the levels in diabetic patients were low in comparison with those of the non-diabetic controls (p-value < 0.001). In favor of our results, Li et al. found a negative association between insulin sensitivity and BDNF levels [13]. This may be due to increased insulin resistance results in disturbed cellular metabolism and increased oxidative stress, which could lead to dysregulation in the release of various neurotransmitters and growth factors, including BDNF. In contrast to our findings, Suwa et al. found that the levels of BDNF were raised among newly diagnosed type 2 diabetics when compared with non-diabetics [14]. These opposing results may be due to differences in sampling techniques or due to resistance developed by BDNF receptors that may coexist with insulin resistance. Serum BDNF levels were also compared among diabetics on the basis of the presence or absence of retinopathy. Similar to our findings, Ola et al. found reduced BDNF levels with developed diabetic retinopathy [9].

Limitations

The limitations of this study included the too-small sample size, being a single-center trial, and financial constraints with a lack of resources. The frequency of subjects with cardiovascular diseases in our study was much lower as compared to other studies, resulting in an inability to detect the effect of these conditions on diabetic retinopathy. A cross-sectional study cannot interpret a causal relationship between serum BDNF levels and retinopathy due to diabetes. Hence, a prospective cohort study is needed to confirm the causal relationship between them.

Strengths

BDNF could be a possible serum biomarker of diabetic retinopathy. It will not only help confirm the diagnosis but will also help grade retinopathy in our population.

## Conclusions

We concluded that there is a significant decline in serum BDNF levels in type 2 diabetics with established retinopathy when compared with the healthy control group. There were decreased serum BDNF levels among patients having long-term diabetic retinopathy, either non-proliferative or proliferative, in comparison with the healthy group as well as diabetics without retinopathy. In addition, we found a positive relationship of higher HbA1c with the development of retinopathy. Hence, serum BDNF levels can be an effective and helpful tool for evaluating diabetic retinopathy.
